# Cost-Effectiveness of Early Treatment with First-Line NNRTI-Based HAART Regimens in the UK, 1996-2006

**DOI:** 10.1371/journal.pone.0020200

**Published:** 2011-05-25

**Authors:** Eduard J. Beck, Sundhiya Mandalia, Gary Lo, Peter Sharott, Mike Youle, Jane Anderson, Guy Baily, Ray Brettle, Martin Fisher, Mark Gompels, George Kinghorn, Margaret Johnson, Brendan McCarron, Anton Pozniak, Alan Tang, John Walsh, David White, Ian Williams, Brian Gazzard, for the NPMS-HHC Steering Group

**Affiliations:** 1 NPMS-HHC Coordinating and Analytic Centre, London, United Kingdom; 2 London School of Hygiene & Tropical Medicine, London, United Kingdom; 3 Imperial College, London, United Kingdom; 4 London Specialised Commissioning Group, London Procurement Programme, London, United Kingdom; 5 Royal Free Hospital, London, United Kingdom; 6 Homerton University Hospital NHS Foundation Trust, London, United Kingdom; 7 London and Barts Hospitals, London, United Kingdom; 8 Edinburgh General Hospital, Edinburgh, United Kingdom; 9 Royal County Sussex Hospital, Brighton, United Kingdom; 10 Southmead Hospital, Bristol, United Kingdom; 11 Royal Hallamshire Hospital, Sheffield, United Kingdom; 12 James Cook University Hospital, Middlesborough, United Kingdom; 13 Chelsea and Westminster Hospital, London, United Kingdom; 14 Royal Berkshire Hospital, Berkshire, United Kingdom; 15 St.Mary's Hospital, London, United Kingdom; 16 Birmingham Heartsland Hospital, Birmingham, United Kingdom; 17 Mortimer Market Centre, London, United Kingdom; Yale University School of Medicine, United States of America

## Abstract

**Aim:**

Calculate time to first-line treatment failure, annual cost and cost-effectiveness of NNRTI versus PIboosted first-line HAART regimens in the UK, 1996–2006.

**Background:**

Population costs for HIV services are increasing in the UK and interventions need to be effective and efficient to reduce or stabilize costs. 2NRTIs + NNRTI regimens are cost-effective regimens for first-line HAART, but these regimens have not been compared with first-line PI_boosted_ regimens.

**Methods:**

Times to first-line treatment failure and annual costs were calculated for first-line HAART regimens by CD4 count when starting HAART (2006 UK prices). Cost-effectiveness of 2NRTIs+NNRTI versus 2NRTIs+PI_boosted_ regimens was calculated for four CD4 strata.

**Results:**

55% of 5,541 people living with HIV (PLHIV) started HAART with CD4 count ≤200 cells/mm3, many of whom were Black Africans. Annual treatment cost decreased as CD4 count increased; most marked differences were observed between starting HAART with CD4 ≤200 cells/mm3 compared with CD4 count >200 cells/mm3. 2NRTI+PI_boosted_ and 2NRTI+NNRTI regimens were the most effective regimens across the four CD4 strata; 2NRTI+NNRTI was cost-saving or cost-effective compared with 2NRTI + PI_boosted_ regimens.

**Conclusion:**

To ensure more effective and efficient provision of HIV services, 2NRTI+NNRTI should be started as first-line HAART regimen at CD4 counts ≤350 cell/mm3, unless specific contra-indications exist. This will increase the number of PLHIV receiving HAART and will initially increase population costs of providing HIV services. However, starting PLHIV earlier on cost-effective regimens will maintain them in better health and use fewer health or social services, thereby generating fewer treatment and care costs, enabling them to remain socially and economically active members of society. This does raise a number of ethical issues, which will have to be acknowledged and addressed, especially in countries with limited resources.

## Introduction

A recent study indicated that the population cost for providing HIV services in the UK has increased considerably and is likely to continue to do so if cost cutting measures are not introduced [1]. One way of reducing cost, is by using the most efficient treatment regimens. The outcome and cost-effectiveness of highly active antiretroviral therapy (HAART) regimens were recently analysed for the period 1996 – 2002. Two nucleoside reverse transcriptase inhibitors comparing non-nucleoside reverse transcriptase inhibitor (2NRTIs+NNRTI) were compared with 2NRTIs and protease inhibitor (PI) containing regimens for first-, second- or third-line treatment for people living with HIV (PLHIV) in the UK [2]. This analysis demonstrated that 2NRTIs+NNRTI regimens were cost-effective regimens for first-, second- or third-line HAART. However, only relatively few patients had been started on PI_boosted_ regimens nor did that analysis investigate differences in the use, cost and outcome of treatment for those patients who started HAART regimens at different CD4 counts. The aim of this study was to investigate the cost-effectiveness of NNRTI containing first-line regimens compared with PI_boosted_ regimens for PLHIV starting at different levels of CD4 count during the period 1996–2006 in the UK.

## Methods

The National Prospective Monitoring System on the use, cost and outcome of HIV service provision in UK hospitals - HIV Health-economics Collaboration (NPMS-HHC) has been monitoring prospectively the effectiveness, efficiency, equity and acceptability of treatment and care in participating HIV units since 1996. Using an agreed minimum dataset, standardised data are routinely collected in clinics and transferred to the NPMS-HHC Coordinating and Analytic Centre (CAC). As the data are transferred in pseudo-anonymized format, patient consent is not required according to the UK Department of Health, which are in line with international guidelines [3]. While ensuring patient and clinic confidentiality, the data are analysed at clinic and aggregate levels: clinic specific analyses remain confidential, while aggregate analyses become public documents [4], [5].

Information on the use of hospital inpatient (IP), outpatient (OP) and dayward services between 1^st^ January 1996 and 31^st^ December 2006, was obtained from computerized information systems from 14 UK hospitals participating in this analysis. HAART became routinely available in the NPMS-HHC clinics in 1996, and subjects who started HAART since then were included in the study. Patients who were transferred from another HIV unit were excluded as it was not possible to establish whether the available HAART combination was indeed their first line regimen. As this study investigated the cost-effectiveness between these regimens when starting at four different CD4 count strata, PLHIV were stratified into four categories based on their CD4 count when starting HAART: ≤100; 101–200; 201–350 and >350 cells/mm3; those with unavailable CD4 count within 4 month before or after starting HAART were excluded from this analysis.

### Use and cost of services

The mean numbers of IP days, OP visits and dayward visits per patient-year (PPY) were calculated for first-line HAART and were stratified by type of regimen. A patient-year was defined as 365.25 days of follow up. The denominator consisted of the total duration of follow up for all patients during the period of first-line treatment with HAART, from when they were first seen till the end of the respective study period if still alive and on first-line HAART, or when they failed first-line HAART or died, or if they were lost to follow up, which ever came first. Numerators were calculated by summing the use of IP, OP or dayward services when on first-line HAART. Mean use of services PPY were calculated using the Poisson regression test for the total population who started first-line HAART as well as for the specified sub-populations disaggregated by CD4 count when starting HAART. The mean use of services was calculated based on a method for calculating the use of services employed in previous studies [1], [2], [6], [7] and summarised by the formula: 
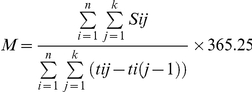
Where n  =  total number of individuals; k  =  day of censoring;S_ij_ =  use of service of individual i at jth day; t_ij_  =  number of days starting and remaining on first-line HAART by CD4 stratum forindividual I; M =  mean of services S per patient-year by CD4 stratum.

First-line HAART failure was defined as any change made to the HAART containing regimen, which included intensification of regimen by adding any anti-retroviral drug to the regimen or swapping the NNRTI or a PI to another anti-retroviral drug class. Dropping a NRTI, NNRTI or PI alone or simplification of ARV combination with no other changes made to the regimen did not constitute treatment failure. Causes for failure included clinical, immunological or virological reasons and others, where adverse effects were the most likely cause [8].

The unit cost for an average IP day was £475, £94 for an OP visit and £384 per dayward visit [9]. IP, OP and dayward costs were obtained by multiplying their mean number of IP days, OP and dayward visits PPY by their respective unit costs for PLHIVs starting at different CD4 counts. The costs generated by the use of services for each of the CD4 categories were added to the costs of HAART, ‘other’ drugs, tests and procedures performed [9]. The costs for the different HAART regimens were weighted average annual prices based on prices negotiated by the London HIV Consortium in 2006 with pharmaceutical companies. The study was performed from a public service perspective [10] and costs for use of services, ‘other’ drugs, tests and procedures performed, were obtained from the 2008 NPMS-HHC report [9]. Costs were calculated in UK pounds (2006 prices) and time to first-line failure and treatment costs were discounted at 3.0% per annum [11].

### Regression Models and Time-to-Treatment Failure

Parametric quantitative data are presented as means with standard deviation (SD) while non-parametric data are presented as medians with inter-quartile range (IQR). Between group comparisons of parametric data were tested using one-way-ANOVA while between group comparisons of non-parametric data were tested using the Kruskal-Wallis test. Qualitative data by CD4 count strata were tested using the χ^2^ test and where appropriate these were adjusted by Yates' correction.

Median and inter-quartile ranges were used to create grouped categories, including a separate category for all variables with missing data. This ensured no degrees of freedom were lost when building multivariable models. Cox's proportional hazards regression models with single variables were initially used to estimate likelihood of treatment failure. All variables found to have a probability of *p*<0.2 in univariate Cox's proportional hazards model were used to build a multivariable model to assess the risk of a particular prognostic variable while controlling for the other variables in the model. The final multivariable model presented was tested for its distributional assumptions using Cox Snell residual plots and adjusted for gender, age, baseline viral load, baseline CD4 count, stage of HIV infection and stratified by year of starting first line HAART for possible confounding or residual effects. Baseline viral load and CD4 cell count were defined as those available 4 months before or after starting first-line HAART and baseline clinical stage was based on the diagnosis within 30 days since starting HAART. Event time was defined as time to treatment failure derived from patient days of follow up. A patient day of follow-up was estimated from start of study period of 1^st^ January 1996, or if entry to cohort came after this date then entry into the cohort date to either the end of the study period of 31^st^ December 2006, failure of HAART regimen, or the last recorded visit during their follow-up.

Analyses of each of four CD4 strata were adjusted for potential confounding or residual effects of sex, age, baseline viral load, baseline CD4 count, stage of HIV infection at start of HAART regimens and stratified by year of starting first-line HAART.

### Survival Function Estimation

After adjusting for confounding and residual variables in the final model, the PROC PHREG in SAS was run with the BASELINE statement to create a new data set with the “survival” function estimates at the event times of each stratum for each list of variables in the final multivariable model [12]. This contained the “survival” function estimates corresponding to the means of the variables in the model for each stratum. The resulting survival function estimates were used to model with event time as a covariate using the least squares maximum likelihood model. The resulting least squares regression model was then used to estimate the extrapolated median and inter quartile ranges (IQR) of time to treatment failure. All analyses were performed using SAS version 9.1.3 statistical software and all significance tests presented are two-tailed.

### Life year gained for first-line HAART regimens

Based on differences in the estimated failure times, the additional life years gained on first-line (LYG-FL) HAART regimens were calculated comparing 2NTRIs+NNRTI regimens with 2NRTIs+PI_boosted_ based on methods used for previous analyses [2], [13], [14]. The incremental cost-effectiveness ratios (ICERs) were calculated using time to first-line failure as outcome measure and based on the following formula [10]:



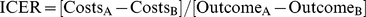



A cost-effectiveness analysis was produced for each of the four CD4 categories.

## Results

### Population characteristics

During the study period, 7600 PLHIV were identified as being on first-line therapy. For 5541 (73%) the CD4 count when starting first-line HAART could be identified. Of the 5541 PLHIVs, 18% failed first-line HAART during the study period; 77% of all PLHIV were men, 59% were Caucasians, 22% Black Africans and 16% were from other ethnic groups. Mean age at start of therapy varied between baseline CD4 count strata from 37.4 (SD 8.9) to 38.2 (SD 8.7) years and 187 PLHIVs were known to be or have been injecting drug users (Table 1).

**Table 1:** Demographic characteristics of PLHIV starting HAART at various CD4 count categories (cells/mm3) and time interval between diagnosis of HIV and starting HAART.

	BaselineCD4 ≤100N = 1547 (%)	BaselineCD4 101–200N = 1503 (%)	BaselineCD4 201–350N = 1815 (%)	BaselineCD4 >350N = 676 (%)	p-value
SexUnknownFemaleMale	5 (0.3)409 (26.4)1133 (73.2)	1 (0.1)347 (23.1)1155 (76.8)	2 (0.1)385 (21.2)1428 (78.7)	2 (0.3)140 (20.7)534 (79.0)	<0.001
Mean Age (SD) at start of therapy	38.2 (8.7)	38.2 (8.4)	37.4 (8.9)	37.0 (8.6)	0.265
Ethnic groupNot availableOtherBlack AfricanCaucasian	163 (10.5)309 (20.0)385 (24.9)690 (44.6)	110 (7.3)264 (17.6)326 (21.7)803 (53.4)	112 (6.2)288 (15.9)323 (17.8)1092 (60.2)	47 (7.0)116 (17.2)103 (15.2)410 (60.7)	<0.001
IDUYesNo	58 (3.7)1489 (96.3)	51 (3.4)1452 (96.6)	56 (3.1)1759 (96.9)	24 (3.6)652 (96.4)	0.816
Median Duration (IQR) since HIV diagnosis to start of first line therapy (years)	0.28 (0.08 TO 4.91)Range: 0.00 to 96.48	1.56 (0.19 TO 5.63)Range: 0.00 to 21.17	2.20 (0.45 to 6.00)Range: 0.00 to 98.92	2.35 (0.42 to 5.88)Range: 0.00 to 20.17	<0.001

The median time between diagnosis of HIV infection and starting HAART for the whole population was 1.6 years (IQR 0.2 to 5.6 years). For those with a CD4 count ≤100 cells/mm3, the time interval between diagnosis of HIV infection was 0.3 years (IQR 0.1 to 4.9), which increased to 2.4 years (IQR 0.4 to 5.9) for those with a CD4 count >350 cells/mm3 (Krukal-Wallis p< 0.001; Table 1). Of all PLHIVs, 55% started HAART with a CD4 count ≤200 cells/mm3. Of those who started with a CD4 count ≤200 cells/mm3, 23% were Black Africans and 49% were Caucasians, which compared with 17% Black African and 60% Caucasians respectively who started with a CD4 count >200 cells/mm3 (X^2^_2_ = 72.6, p<0.001; Table 1).

### Estimated time to first-line treatment failure

PLHIV on 2NRTI's + PI_boosted_ or 2NRTI's + NNRTIs were less likely to fail than those that started on other combinations. Across all CD4 strata, estimated median time to first-line failure for those who started on 2NRTIs + PI_boosted_ was 18.5 years (IQR 9.0 to 28.1) compared with an estimated median of 13.9 years (IQR 6.3 to 19.9) for those starting on 2NRTI's + NNRTI.

When stratified at a CD4 count of 200 cells/mm3, results were similar for those obtained for the total population, with the 2NRTIs + NNRTI and 2NRTI+PI_boosted_ regimens being most effective compared with other regimens. For PLHIV starting on 2NRTI's + PI_boosted_ with CD4 counts ≤200 cells/mm3, estimated median time to first-line failure was 18.5 years (IQR 9.0 to 28.1) compared with 14.7 years (IQR 6.6 to 22.9) for PLHIV starting on 2NRTIs + NNRTI regimens (Hazard ratio = 0.5; 95%CI 0.32 to 0.78, p = 0.002). For those PLHIV starting on 2NRTI's + PI_boosted_ with a CD4 counts >200 cells/mm3, estimated median time to first-line failure was 13.1 years (IQR 6.3 to 19.9) compared with 13.9 years (IQR 6.5 to 21.3) for those starting on 2NRTIs + NNRTI regimens (Hazard ratio = 0.9; 95%CI 0.57 to 1.41, p = 0.642).

**Figure 1:**
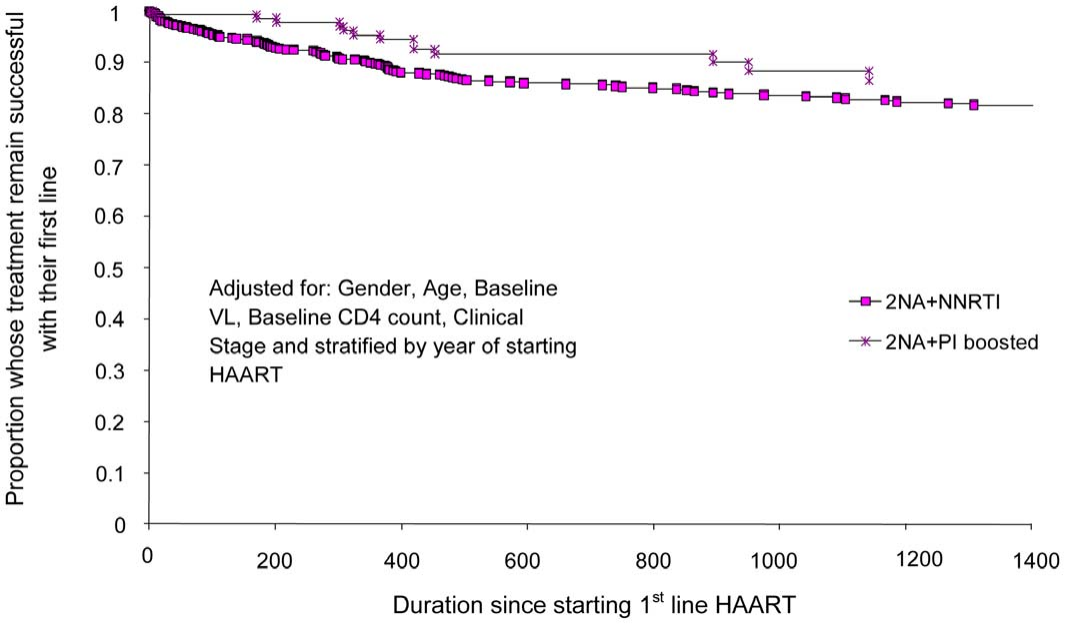
Proportion of people starting HAART at CD4 count ≤100 cells/mm3 who failed first-line therapy and time to treatment failure (days) comparing 2NRTIs+NNRTI with 2NRTIs+PI_boosted_ first-line regimens.

When CD4 counts were stratified into four strata, the 2NRTIs + PI_boosted_ regimens had a longer estimated time to first-line failure compared with 2NRTIs + NNRTI regimens only for those PLHIV who started HAART with a CD4 count between 101–200 cell/mm3. For the other three strata, the 2NRTIs + NNRTI regimens had similar or longer estimated times to first-line failure (Table 2; Figures 1–4). In addition to the impact of the antiretroviral drugs, women, younger people and those with an AIDS diagnosis were all more likely to fail first-line therapy (Table 2).

**Figure 2 pone-0020200-g002:**
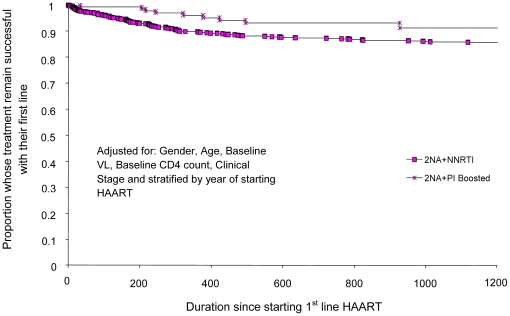
Proportion of people starting HAART at CD4 counts 101 – 200 cells/mm3 who failed first-line therapy and time to treatment failure (days) comparing 2NRTIs+NNRTI with 2NRTIs+PI_boosted_ first-line regimens.

**Figure 3 pone-0020200-g003:**
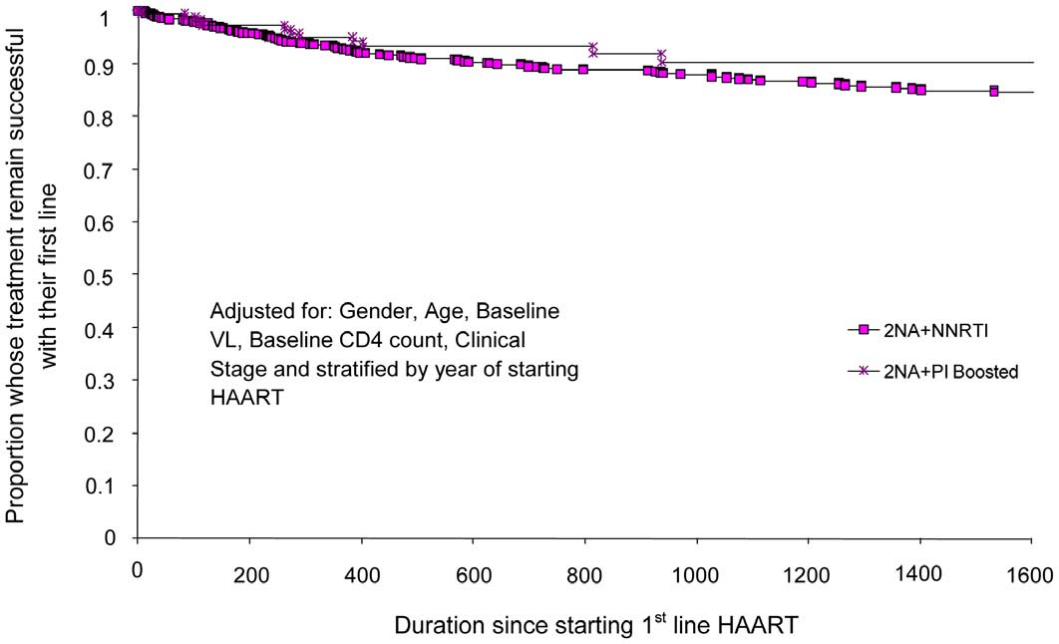
Proportion of people starting HAART at CD4 count 201 – 350 cells/mm3 who failed first-line therapy and time to treatment failure (days) comparing 2NRTIs+NNRTI with 2NRTIs+PI_boosted_ first-line regimens.

**Figure 4:**
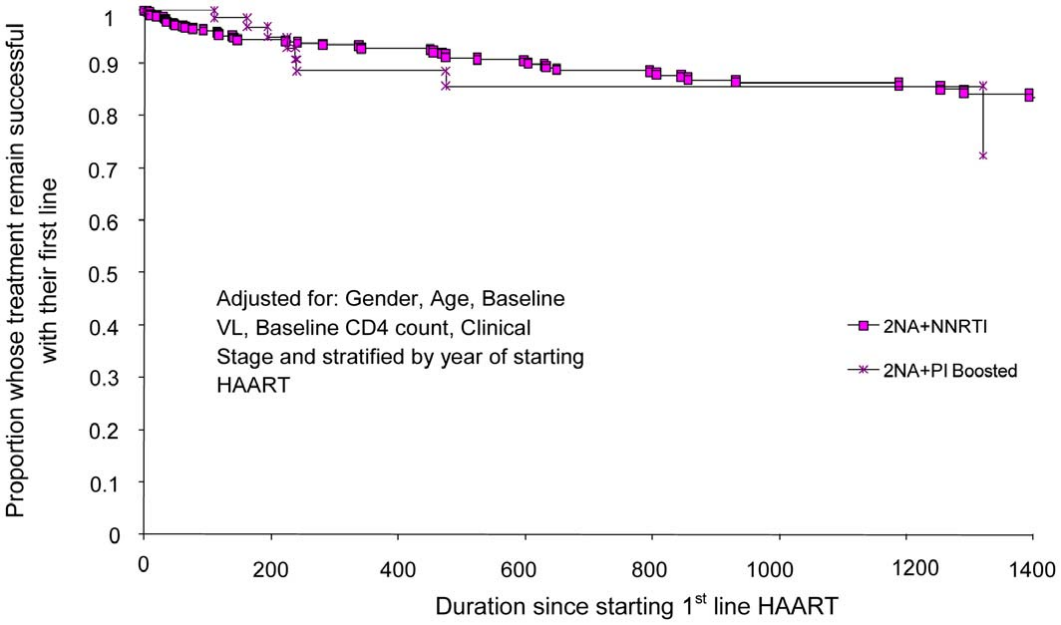
Proportion of people starting HAART at CD4 count >350 cells/mm3 who failed first-line therapy and time to treatment failure (days) comparing 2NRTIs+NNRTI with 2NRTIs+PI_boosted_ first-line regimens.

**Table 2:** Multivariate Cox's proportional hazards regression model of independent predictors of treatment failure for first-line HAART, adjusted for age, sex, baseline clinical status, viral load and CD4 count, and stratified by year of starting first-line HAART.

Table 2	Baseline CD4 ≤100N = 1547			Baseline CD4 101–200*N = *1503			Baseline CD4 201–350N = 1815			Baseline CD4 >350*N = *676		
	Failed first-line therapyN* = *331			Failed first-line therapyN = 255			Failed first-line therapyN* = *278			Failed first-line therapyN = 111		
Variables	HR	95% CI	Score statisticp-value	HR	95% CI	Score statisticp-value	HR	95% CI	Score statisticp-value	HR	95% CI	Score statisticp-value
Sex:FemaleMale	1.171	(0.89 to 1.53)	0.256	1.591	(1.19 to 2.13)	0.002	1.411	(1.06 to 1.88)	0.020	1.961	(1.21 to 3.18)	0.007
Age	0.99	(0.97 to 0.99)	0.035	0.99	(0.97 to 1.01)	0.174	1.00	(0.99 to 1.01)	0.816	0.98	(0.96 to 1.01)	0.147
Clinical statusAIDSNon AIDS	1.321	(1.04 to 1.67)	0.023	1.541	(1.19 to 2.00)	0.001	1.321	(1.01 to 1.72)	0.041	1.091	(0.69 to 1.71)	0.719
First line regimensOther2NRTIs+PI2NRTIs+2PI2NRTIs+PIboosted2NRTIs+NNRTI	2.181.491.320.541	(1.56 to 3.03)(1.06 to 2.09)(0.18 to 2.09)(0.30 to 0.97)	<0.0010.0200.7850.037	2.182.183.760.551	(1.55 to 3.07)(1.51 to 3.16)(1.03 to 13.80)(0.29 to 1.05)	<0.001<0.0010.0460.068	1.542.170.740.861	(1.02 to 2.31)(1.56 to 3.01)(0.10 to 5.35)(0.49 to 1.50)	0.040<0.0010.7610.597	1.491.730.001.511	(0.79 to 2.80)(1.09 to 2.74)(-)(0.69 to 3.28)	0.2180.0210.9860.303
Extrapolated and estimated median time (IQR) to failure for first-line HAART regimens (in days)												
Other regimens2NRTIs+PI2NRTIs+2PI2NRTIs+PIboosted2NRTIs+NNRTI	2528 (1118 to 3938)1791 (770 to 2811)1485 (742 to 2227)4218 (2086 to 6350)4707 (2097 to 7317)			2501 (1143 to 3859)2394 (1094 to 3694)231 (581 to 391)7051 (3425 to 10677)5600 (2522 to 8678)			2640 (1295 to 3986)2676 (1211 to 4141)604 (302 to 906)4607 (2266 to 6948)5211 (2460 to 7962)		1573 (845 to 2302)2678 (1244 to 4112)Not possible to estimate2324 (1112 to 3536)5072 (2362 to 7783)			

### Annual cost of treatment and care

Those PLHIV with CD4 counts >200 cells/mm3 had fewer IP days compared with those starting HAART with a CD4 count ≤200 cells/mm3. When analyzed across the four CD4 strata, the mean number of IP days was highest for those PLHIV who started HAART with ≤100 cells/mm3 and IP days decreased as CD4 count increased (Table 3). Similar differences were observed for the mean number of OP and dayward visits, though less pronounced than for IP days. Across all CD4 strata, PLHIV on 2NTRIs+NNRTI used fewer services than those who started on 2NTRIs+PI_boosted_ regimens (Table 3).

**Table 3:** Mean number of inpatient Days, outpatient and dayward visits for PLHIV on different first-line HAART regimens, annual cost for different HAART regimens and cost-effectiveness analyses comparing 2NRTIs+NNRTI and 2NRTIs+PI_boosted_ for different CD4 count categories (2006 UK prices).

	Baseline CD4 ≤100N = 1547	Baseline CD4 101-200N* = *1503	Baseline CD4 201-350N = 1815	Baseline CD4 >350N* = *676
**Mean number of Inpatient Days for different HAART regimens**				
2NRTIs+PI2NRTIs+2PI2NRTIs+PI_boosted_2NRTIs+NNRTI	8.702.346.073.47	4.426.441.891.72	1.603.212.571.14	2.012.891.741.26
**Mean number of Outpatient Visits for different HAART regimens**				
2NRTIs+PI2NRTIs+2PI2NRTIs+PI_boosted_2NRTIs+NNRTI	12.4710.8611.388.95	11.6512.2210.247.33	10.764.110.598.11	10.8710.7411.358.56
**Mean number of Dayward Visits for different HAART regimens**				
2NRTIs+PI2NRTIs+2PI2NRTIs+PI_boosted_2NRTIs+NNRTI	1.440.000.610.14	1.530.000.250.09	0.180.000.140.11	1.550.000.360.13
**Annual cost of Treatment and care for different HAART regimens**				
2NRTIs+PI2NRTIs+2PI2NRTIs+PI_boosted_2NRTIs+NNRTI	£25,751£27,306£24,556£20,730	£23,679£29,381£22,327£19,722	£14,816£20,158£15,721£12,605	£15,544£20,633£15,478£12,713
**Cost-effectiveness of NNRTI versus PI_boosted_ Regimens**				
2NRTIs+NNRTIversus2NRTIs+PI_boosted_	Saves £35,194 per annum of first line HAART	-----------	Saves £37,529 per annum of first line HAART	£10,165 per added year of first line HAART
2NRTIs+PI_boosted_versus2NRTIs+NNRTI	---------	£35,361 per added year of first line HAART	---------------	--------------

For all CD4 strata the annual treatment and care costs of PLHIV on 2NRTIs + NNRT regimens were less compared with those on 2NRTIs + PI_boosted,_. While annual costs decreased with increasing CD4 count, the greatest difference in annual costs was observed between those people who started HAART with a CD4 count ≤200 cells/mm3 compared with those with a CD4 count >200 cells/mm3 (Table 3).

### Cost-effectiveness of NNRTI versus PI_boosted_ regimens

Both NNRTI and PI_boosted_ regimens were effective first-line regimens. However 2NRTIs+NNRTI regimens were cost-saving for PLHIV starting on HAART with CD4 counts ≤100 cells/mm3 and between 201–350 CD4 cells/mm3. For those starting HAART with a CD4 count >350 cells/mm3, the cost per additional life-year gained in first-line therapy on 2NRTIs+NNRTI was £10,165; for those who started with CD4 counts between 101–200 cells/mm3, the cost of an additional life-year gained on 2NRTIs+PI_boosted_ regimens was £35,361 (Table 3).

## Discussion

The 2NRTI + NNRTI and 2NRTI + PI_boosted_ regimens were the most effective first-line HAART regimens. The annual treatment costs were less for those managed with 2NRTIs + NNRTI compared with 2NRTIs + PI_boosted_. Not only were drug cost less for 2NRTIs + NNRTI regimens, these patients also used fewer hospital services, resulting in lower annual treatment costs.

For three of the four CD4 strata, 2NRTIs + NNRTI regimens were either cost-saving or cost-effective compared with 2NRTIs + PI_boosted_ regimens. Only when HAART was started at a CD4 count between 101–200 cells/mm3 did 2NRTIs + PI_boosted_ regimens have a longer time-to-first-line failure but at a cost of £35,361 per additional first-line life-year gained. Similarly, for those who started 2NRTIs + PI_boosted_ regimens with CD4 count ≤200 cells/mm3, the cost per life-year-gained was £39,533 compared with 2NRTIs + NNRTI regimens, while 2NRTIs + NNRTI regimens were cost saving compared with 2NRTIs + PI_boosted_ regimens with CD4 counts >200 cells/mm3 [15]. Both £35,361 and £39,533 costs per additional first-line life-year gained are above the £35,000 cut-off point, at which NICE considers interventions not to be cost-effective [16].

While these analyses were based on a large number of subjects followed-up over years, the analyses have limitations. Firstly, the data were collected in 14 sites, 7 London and 7 out-of London hospitals, but 91% of patients contributing to this study, were seen in London sites. Secondly first CD4 count when starting HAART could not be retrieved for all those who were identified as starting first-line and 27% of patients had to be excluded. Thirdly, the number of PLHIV starting on HAART with CD4 count >350 cells/mm3 were considerably less than those starting with a CD4 count ≤350 cells/mm3. This may increase with changing clinical practice for initiating HAART and longer follow-up, but given the similarity of results with those starting with CD4 count between 201–350 cells/mm3, the results may not change. Fourth, the data available for operational research are by definition observational data [17]. While results were adjusted for a number of key potential confounders, some residual confounding may have remained and affected the results.

Despite these limitations, lessons can be drawn from these analyses. The annual cost of treatment and care were less for those starting HAART with higher CD4 counts, partly due to less inpatient care. While a gradual decrease in annual treatment costs are observed with increasing CD4 count, the most marked cost differences were observed between those who start with a CD4 count ≤200 cells/mm3 compared with those with a CD4 count >200 cells/mm3. Recent Canadian and US studies produced similar results, where PLHIV with CD4 counts >200 cells/mm3 used fewer health services and the annual cost of services was less than for PLHIV who had a CD4 count ≤200 cells/mm3 [18], [19].

Based on the data presented, starting with a first-line NNRTI regimen when CD4 count drops below 350 cells/mm3 currently is the optimum first-line strategy [20]–[22] provided no specific contra-indications exist. Current BHIVA and the new WHO guidelines reflect this by recommending starting HAART when the CD4 count drops below 350 cells/mm3 [23], [24]. Until recently US guidelines recommended a similar cut-off point to start HAART [25], but the latest guidelines recommended starting when CD4 count drops <500 cells/mm3 [26]. Apart from the fact that these last guidelines were not unanimously adopted, these changes have also been questioned on the basis that the available evidence is currently insufficient to determine if the adherence challenges and long-term side-effects of early antiretroviral treatment are outweighed by reduced risk of illness conferred by these medicines when starting with a CD4 count <500 cells/mm3 [27]. While a recent US study reported that hospitalization rates for those on HAART with a CD4 count <350 cells/mm3 did not differ significantly from those with a CD4 count ≥350 cells/mm3 [28], more definitive answers to these questions will hopefully be provided by the START study [29].

It remains a sobering finding that 55% of PLHIVs started HAART with a CD4 count ≤200 cells/mm3, a disproportionate number of whom were Black Africans compared with those who started HAART with CD4 counts >200 cells/mm3. Having more PLHIVs starting HAART with a CD4 count <350 cells/mm3 will increase the number of people receiving HAART, which will initially add to the population cost of service provision [1]. Healthcare systems in many high-, middle- and low-income countries are already under considerable financial strain, which has been exacerbated by the global economic downturn [30]. However, starting PLHIVs on these cost-effective regimens earlier, will maintain them in better health, resulting in them needing to use fewer health or social services, thereby generating fewer treatment and care costs, enabling them to remain socially and economically active members of society and reducing population costs in the medium- or long-term.

Some workers in the field maintain that through ‘test and treat early’ strategies we may be able to eliminate the HIV pandemic [31]. While the costs of such a strategy have been questioned [32] and it is questionable whether this goal is achievable with current treatment [33], the findings presented in this study provide social, financial and economic arguments which strengthen the case for HIV testing and earlier treatment strategies [34]. A recent modelling study from the US suggests that expanding HIV testing and starting early treatment with ART provide the greatest health benefits and are cost-effective, although the authors concluded that these measures in themselves are not sufficient to markedly reduce the US epidemic and this also needs to be complemented by successful behavioural strategies to stop people becoming newly infected with HIV [35].

However stigma and discrimination remain strong disincentives for people to come forward to be tested, especially if it involves hard-to-reach key populations, so testing campaigns need to be coupled to measures to ensure the confidentiality and security of such personal information [2]. Furthermore, in countries with limited resources this raises a number of ethical issues: should those with most severe disease continue to be the first to receive antiretroviral therapy? Should those with higher CD4 counts be treated first, as they generate fewer costs by using fewer resources and thereby enabling more PLHIVs to be treated or should PLHIV receive HAART on a ‘first come and first-serve basis’? In addition the assumption that antiretroviral treatment is for life as accepted in high income countries [36] may also be questioned. It is neither the intention nor the place of this paper to provide answers to these questions as countries will need to develop and implement their own context specific solutions. However, if these broader aspects are not considered and successfully addressed, early ‘test and treat’ may turn out to be more of a ‘trick’ than a ‘treat’.
